# Unlocking the power of virtual networking for early-career researchers

**DOI:** 10.7554/eLife.96381

**Published:** 2024-03-19

**Authors:** Cellas A Hayes, Jordan T Moore, Colwyn A Headley, Almarely L Berrios-Negron, W Marcus Lambert

**Affiliations:** 1 Department of Epidemiology and Population Health, School of Medicine, Stanford University Stanford United States; 2 Department of Materials Science and Engineering, Stanford University Stanford United States; 3 Department of Neurology, School of Medicine, Stanford University Stanford United States; 4 Stanford Cardiovascular Institute, School of Medicine, Stanford University Stanford United States; 5 School of Behavioral and Brain Sciences, Ponce Health Sciences University Ponce United States; 6 School of Public Health, State University of New York Downstate Health Sciences University Brooklyn United States

**Keywords:** equity, diversity, inclusion, early-career researchers, mentoring, networking, professional development, Human, None

## Abstract

Many successful researchers in the biomedical sciences have benefitted from mentors and networks earlier in their career. However, early-career researchers from minoritized and underrepresented groups do not have the same access to potential mentors and networks as many of their peers. In this article we describe how ‘cold emails’ and social media platforms – notably Twitter/X and LinkedIn – can be used to build virtual networks, and stress the need to invest in maintaining networks once they have been established.

## Introduction

The journey from being an undergraduate student to having a career in biomedical research is more likely to be successful if you have one or more mentors – experienced scientists who you can turn to for help and advice. Mentors typically advise trainees on specific tasks (such as applying for jobs and grants/fellowships, designing experiments, and getting promoted), and also on soft skills such as managing people and time, and may even be willing to write you letters of recommendation for various opportunities based on your career stage. It is also a good idea for an early-career researcher to build a network of other researchers who are at similar career stages, and have similar research interests, to enhance peer-to-peer support.

However, some early-career researchers have much less access to potential mentors and networking opportunities than others. In particular, those working at smaller research institutions – such as historically black colleges and universities, other minority serving institutions, and primarily undergraduate serving institutions – are disadvantaged when it comes to finding mentors and building networks. Similarly, when an underrepresented minority early-career researcher moves to a large research-intensive university and wants to seek a mentor who has a similar background, their options are limited, with just 7% of faculty members being Black and 6% being Hispanic (National Center for Education Statistics, 2023). And when it comes to mentoring and networking more generally, there is still considerable room for improvement, including formal training for postdocs and their mentors (Gangrade and Lambert, 2024; see also Box 1 about the National Research Mentoring Network).

Box 1. **The National Research Mentoring Network.**

The National Research Mentoring Network (NRMN) is part of a broader initiative called the Diversity Program Consortium (DPC), which was set up by the National Institutes of Health in the US to enhance the diversity of the NIH-funded workforce. The aim of the NRMN is to reshape the biomedical research workforce by emphasizing the value of mentoring and networking, especially for individuals from historically underrepresented backgrounds (Javier et al., 2021; Sorkness et al., 2017). Individuals at all career stages can join the NRMN platform and be connected to potential mentors or mentees. However, the resources in the online portal built by the NRMN seem to be underutilized relative to other resources such as Twitter/X and LinkedIn (Mervis, 2017; Mervis, 2020).

**Figure 1.**
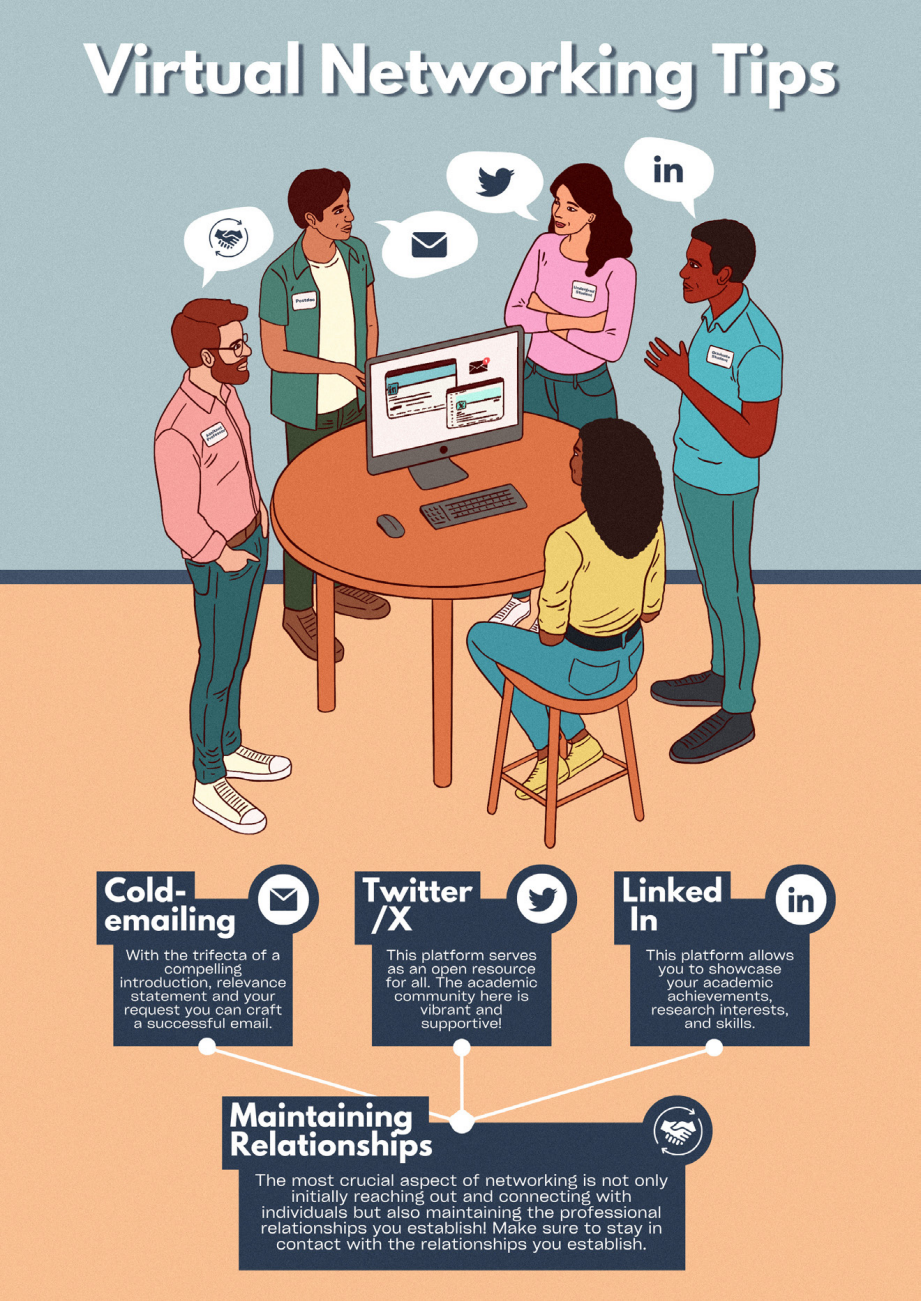
Virtual networking tips for early-career researchers. An early-career researcher can use a combination of cold emailing, Twitter/X and LinkedIn to build their scientific network. However, once you have added someone to your network, it is essential that you also make an effort to maintain the relationship.

In this article, we outline how virtual networking – in the form of cold emailing and social media (notably Twitter/X and LinkedIn) – can be a powerful tool for addressing and overcoming these disparities and systemic barriers (Figure 1). We discuss how to make the most of these virtual opportunities, and stress the need to nurture and sustain these relationships over time. Although most of the examples are taken from the United States, where the authors are based, we feel that our advice is also applicable at the international level.

## Building and maintaining a network

A good place to start is with the people you already know – your existing network and current mentors. Maybe you just want to have a bigger network, or you want to establish a connection with someone who has particular skills or knowledge that would be helpful. In the latter case we suggest that you establish if anyone in your existing network already has these skills or knowledge, or if they know anyone who has them. This advice might sound like common sense but, in our experience, it is often ignored or disregarded by early-career researchers. Likewise, we would also advise against becoming fixated with finding ‘the expert’ in a particular area, as such people are almost always extremely busy. That said, one of the advantages of social media is that it allows you to establish connections with world experts, even if you cannot add them to your personal network.

### How to write a cold email

The thought of sending a ‘cold email’ to someone can be intimidating and anxiety-inducing, but it is quite common in both academia and industry. Indeed, many academic researchers are enthusiastic about mentoring and connecting with emerging scientists beyond their primary affiliations (usually because they have benefited from being mentored in this way and are keen for others to also benefit).

In our experience, there are three key components of a good cold email – the introduction, the relevance statement, and the request – and we will go through these one by one.

**The introduction:** Start by briefly introducing yourself (including your institution/location, training level, and future aspirations), and then try to forge a connection with the person you are writing to. Ways to forge a connection could include having read a preprint or paper by this person, or having heard a talk by them at a conference. The introduction should typically be 2–3 sentences long.

**The relevance statement:** Discuss something about the person that is relevant to your conversation, such as their research or a program they are involved in. The relevance statement needs to demonstrate a keen understanding of the work/interests of the person that you are writing to, and to make it clear on how that aligns with your own work/interests. The relevance statement should typically be 3–5 sentences long.

**The request:** Clearly state what you are seeking, which might be mentorship, advice or guidance, or a potential collaboration. And if you are requesting a meeting, conclude your email by proposing specific days and times you are available and offering to send them a calendar invite with the Zoom link. The request should typically be 2–4 sentences long.

Sending a cold email may seem daunting at first, but it will become less intimidating with experience.

### Connecting on social media: Twitter/X

Social media is an excellent way of finding out about new papers, fellowship opportunities, job openings, and virtual events. It is also a powerful tool for virtual networking, even if you are not comfortable posting material on a regular basis. Here we discuss two social media platforms – Twitter/X and LinkedIn. Other platforms to consider include Bluesky, Instagram, and TikTok (Hines and Warring, 2019; Rein, 2023).

From our personal experience, the academic community on Twitter/X is vibrant and supportive, making it a fantastic place to expand your network beyond your institution, despite the recent change of name and ownership. As we describe below, many scientific groups and organizations are all still active on the platform.

At a basic level, Twitter/X can be used to find out about various opportunities (such as grants, fellowships and professional development activities) at funding agencies, foundations, and scientific societies. Most scientific organizations are on Twitter/X, though researchers should be aware that larger organizations often have more than one account: the National Institutes of Health (NIH) in the US, for example, has an overall account (@NIH), a number of accounts for the different institutes with the NIH, and still more accounts on specific themes and topics (such as @NINDSDiversity and @NIH_LRP). Most journals are also on Twitter/X. Searching for hashtags – such as #PhDVoice and #PostdocJobs – is also a good way to find content of interest to you: see this google doc for extensive lists of scientific hashtags.

Various affinity groups have taken advantage of Twitter/X over the past four years, such as Black in Neuro (@BlackInNeuro), Black In X (@blackinxnetwork), Black In Chem (@BlackInChem), Latinas In Neuro (@LatinasInNeuro), the Society for the Advancement of Chicanos/Hispanics & Native Americans in Science (@SACNAS), and the Annual Biomedical Research Conference for Minoritized Scientists (@ABRCMS).

A number of researchers are also active on Twitter/X, often posting lengthy ‘threads’ that summarize the main findings of their most recent papers: the online discussions that are started by these threads are often compared to a virtual journal club. Posting about your own research – including posts about the challenges you encounter – is likely to increase interest in your work and lead to new connections with other researchers in your field. Posting about your own research can be daunting, especially with the fear of getting scooped; thus, it is more common to create smaller threads on publications and preprints. When other researchers post similar threads about their own work, it is perfectly OK to ask questions within the thread, or to retweet with your question and ask others about their thoughts, or to privately message the author with your questions.

An unconventional method of using both Twitter/X and cold emails is to search for researchers who have posted about being successful (or unsuccessful) when applying for a research grant or fellowship, and then contact them by email or direct messaging. Researchers who have successfully applied for a grant or fellowship are sometimes willing to share their applications and/or to give feedback on draft applications. One of the present authors (CAH) used this approach when he was a graduate student at the University of Mississippi: “I searched for F31 and found individuals who had received the fellowship within the last year. From there, I privately messaged them introducing myself and letting them know I was writing an application, but no one in my lab or institution had done so before. I received five different applications. Using these applications as a guide, I became the first trainee at the University of Mississippi to receive a F31 in 37 years and the first black trainee to receive the award ever at the institution. I have since used this same approach as a postdoctoral fellow at Stanford when applying for other awards.” Another of the authors (JTM) has also employed this approach.

### Connecting on social media: LinkedIn

LinkedIn is a platform that serves as a virtual resume and a professional portfolio, allowing you to showcase your academic achievements, research interests and skills. Much like Twitter/X, you can use LinkedIn to learn about career opportunities and to connect with other researchers and professionals. LinkedIn also provides the opportunity to join groups and engage in discussions that are relevant to your work, and it is particularly useful for exploring career opportunities beyond academia (Davis et al., 2020).

If you decide to join LinkedIn, we recommend that you do the following: (i) create a robust profile that highlights your strengths and accomplishments: YouTube contains lots of advice on how to make a “great LinkedIn profile” and how to build a “personal brand” on the platform; (ii) follow companies and organizations of interest to stay informed about job opportunities and industry developments; (iii) actively engage with posts by liking, sharing, and commenting, demonstrating your presence and interest to potential connections.

Whether you are pursuing a career in academia, industry or elsewhere (such as jobs in science communication or science policy), a well-maintained LinkedIn profile will make you more discoverable to potential employers, and will also help to expand your network.

### Maintaining your network

Once you have added someone to your network, it is important to maintain the relationship. Sending periodic updates on your research progress, achievements and publications is an effective way to keep your network informed and engaged: we have found that every six months is a good frequency for such email updates for individuals within your network. These emails can also be centered around conferences and meetings as researchers often use such events as opportunities to connect with their external mentees. And if someone in your network is unable to attend in person, they may introduce you to members of their laboratory who will be at the meeting, further expanding your network. (Please see Beasley et al., 2024 for advice on effective networking at in-person events such as conferences).

Pertaining to mentors, we have found it useful to have quarterly Zoom calls with mentors who are not based at our home institutions. It is also important to realise that networking is not just about what others can do for you, it is about building mutually beneficial relationships. Therefore, you should ask your mentors about their own work, and also thank them for their support and advice.

There are other approaches to virtual networking, such as virtual events, and contacts made at these events which should also be maintained if you presume the relationship to be beneficial (Iyengar et al., 2022).

## Concluding remarks

Virtual networking can bring many benefits to early-career researchers. Some senior researchers argue that early-career researchers simply do not have the time for activities like this, and that their time would be better spent doing research and writing papers. However, the present authors – who work in a range of different fields and roles – feel that, based on our own experiences with these approaches, the positives far outweigh the negatives, and that virtual networking has the potential to help early-career researchers, especially those from underrepresented minorities, to make progress in their careers.
